# Validation of the Nine Item Avoidant/Restrictive Food Intake Disorder Screen (NIAS) in a Flemish Sample of Community Adults

**DOI:** 10.3390/nu18213498

**Published:** 2026-10-24

**Authors:** Laurence Claes, Dirk Smits, Lore Vankerckhoven, Glenn Kiekens, Koen Luyckx

**Affiliations:** 1 Faculty of Psychology and Educational Sciences, Katholieke Universiteit Leuven, 3000 Leuven, Belgium; dirk.smits@odisee.be (D.S.); lore.vankerckhoven@kuleuven.be (L.V.); g.kiekens@tilburguniversity.edu (G.K.); koen.luyckx@kuleuven.be (K.L.); 2 Child & Youth Institute, Katholieke Universiteit Leuven, 3000 Leuven, Belgium; 3 Faculty of Medicine and Health Sciences, University of Antwerp, Wilrijk, 2610 Antwerp, Belgium; 4 Research Department, Odisee University of Applied Sciences, 1000 Brussels, Belgium; 5 Center for Contextual Psychiatry, Katholieke Universiteit Leuven, 3000 Leuven, Belgium; 6 Department of Medical and Clinical Psychology, Tilburg University, 5037 AB Tilburg, The Netherlands; 7 Educational Unit for Professional Training and Service in the Behavioural Sciences (UNIBS), University of the Free State, Bloemfontein 9301, South Africa

**Keywords:** NIAS, ARFID, reliability, validity, adults

## Abstract

**Background/Objectives**: Avoidant/Restrictive Food Intake Disorder (ARFID) is characterized by restricted food intake with significant physical and/or psychosocial consequences. ARFID comprises three subtypes: sensory-based food avoidance, lack of interest in eating, and fear of aversive consequences of eating. The Nine Item ARFID Screen (NIAS) was developed to assess these three ARFID subtypes. This study evaluated the psychometric properties of the Dutch NIAS in a Flemish adult sample. **Methods**: A community sample of 424 adults (18–65 years) completed the Dutch NIAS and self-report measures assessing eating disorder symptoms, psychological symptoms, and identity functioning. The factor structure, internal consistency, and measurement invariance of the NIAS scales were examined, as were the associations between the NIAS scales and psychological symptoms. **Results**: Confirmatory factor analysis supported the three-factor structure of the Dutch NIAS. The three subscales showed good internal consistency (all α/ω ≥ 0.82). Partial scalar invariance across BMI categories was supported after freeing Item 7, whereas scalar invariance across gender and age groups was not established. NIAS subscale scores were significantly positively related to eating disorder symptomatology, anxiety, depression, somatic symptoms, and identity diffusion, and they were negatively associated with BMI, identity synthesis, and self-esteem. **Conclusions**: The Dutch NIAS demonstrates good reliability and factorial validity for assessing ARFID-related symptoms in the younger adult subgroup (<40 years) across gender and weight categories. However, the observed differences in measurement invariance, particularly across age groups, warrant further investigation in larger and more diverse samples.

## 1. Introduction

Avoidant/Restrictive Food Intake Disorder (ARFID) is a feeding and eating disorder formally introduced in the Diagnostic and Statistical Manual of Mental Disorders, Fifth Edition (DSM-5) [1] and retained in the DSM-5-TR [2]. ARFID can be diagnosed at any age when inadequate nutritional or caloric intake results in weight loss, nutritional deficiency, dependence on nutritional supplements, and/or significant interference with psychosocial functioning [2,3,4]. Unlike anorexia nervosa and bulimia nervosa, AFRID is not associated with body image disturbance or an intense fear of weight gain [2]. ARFID can be divided into three phenotypes based on the motivations behind the food restriction: (1) food avoidance or restriction based on the sensory characteristics of food (e.g., color, smell, or texture), (2) avoidance driven by concerns about negative consequences of eating, such as choking or vomiting, and (3) a lack of interest in eating or food, often accompanied by low appetite [2]. Evidence-based treatment generally involves phenotype-specific cognitive behavioral therapy: food exposure for the sensory-sensitive subtype, exposure and cognitive restructuring for fear-based avoidance, and structured meal planning with behavioral activation for the low-interest subtype [5]. Family involvement and nutritional support are often included across phenotypes [5].

Nicholls-Clow et al. [6] were the first to examine the prevalence of ARFID using a meta-analysis (including 26 studies, *n* = 122,861 participants, age range: 4 months–94 years). Their meta-analysis showed a prevalence of 11.14% (95% CI 8.16–14.5%) and of 4.51% (95% CI 0.7–10.68%) if only high-quality studies were included in a mixed sample of children/adolescents and adults. Additionally, the study showed that the prevalence was slightly higher among male children/adolescents and adults compared to female children/adolescents and adults and among children/adolescents versus adults [6]. Additionally, Brownlow et al. [7] estimated the prevalence rates of ARFID in a UK and USA representative adult sample (50% females) by means of the Nine Item ARFID Screen (NIAS) and not controlling for other eating disorders. Among these, 26% of the population screened positive for ARFID. Prevalence estimates were significantly higher in females (29.6%) compared to males (22.1%) and differed by age: 18–39 years (31.6%), 40–64 years (25.0%), and ≥65 years (16.1%) [7].

Accurate identification of ARFID is crucial due to the risk of serious complications and the requirement for specialized treatment [4]. ARFID is commonly associated with higher rates of underweight, mood problems, somatic symptoms, disorders of gut–brain interaction, reduced psychological and physical quality of life, and increased healthcare utilization [7,8]. Therefore, having a psychometrically robust screening measure for ARFID is essential to ensure accurate identification of potential causes across populations [4].

One such tool used to assess ARFID is the Nine Item ARFID Screen (NIAS) [8]. Participants rate the 9 items on a scale ranging from 0 (strongly disagree) to 5 (strongly agree) with higher scores reflecting greater symptom severity on three subscales (picky eating, lack of appetite, fear) and a total score [8,9]. Cutoff scores of ≥10 on the picky eating subscale, ≥9 on the appetite subscale, and/or ≥10 on the fear subscale indicate a probable ARFID diagnosis when used alongside the Eating Disorder Examination Questionnaire (EDE-Q) [10] to differentiate ARFID from other eating disorder presentations [9]. Although Burton Murray et al. [9] proposed empirically derived NIAS screening cutoffs, these thresholds have not yet been extensively validated in independent clinical reappraisal studies using blinded structured diagnostic interviews. Therefore, they should be interpreted as screening indicators rather than diagnostic cutoff scores.

The NIAS, developed in English, has been translated (and psychometrically validated) in multiple languages, including Arabic [11], Chinese [12], Dutch (not validated yet) [13], Farsi [14], French [15], Polish (parent report) [16], Spanish [17], and Turkish (parent version [18], self-report [19]). It has also been validated across different age groups [8,9,20], genders (e.g., transgender individuals) [3], and in both healthy and clinical populations (e.g., autistic individuals; veterans) [4,21]. Confirmatory factor analyses in the aforementioned studies provided evidence for three factors of the NIAS. The NIAS subscales demonstrated high internal consistency, test–retest reliability, measurement invariance across age, gender, weight groups, clinical/healthy groups, and convergent/discriminant validity with other measures of picky eating, appetite, fear of negative consequences, and (psycho)pathology [8]. For a review of the different validation studies in adults (see Appendix A).

However, to our knowledge, no studies have examined the Dutch version of the NIAS in a large adult community sample in Flanders (Belgium), limiting our ability to screen for and describe ARFID in the population. To address this gap in the literature, we addressed four research questions within a Flemish adult community sample (18–65 years). First, we tested the three-factor structure of the NIAS and a higher-order model with one latent construct (i.e., NIAS-total), as well as the correlations among the latent factors. Based on previous findings, we expected to find support for a three-factor structure of the NIAS [4,8,11,12,21]. We further hypothesized a second-order model in which each of the subscales loaded on one higher-order latent factor (i.e., NIAS-total). Many studies have used the NIAS total score but none of these studies have tested whether the factor structure with one higher-order factor fitted the data well, which fills a gap in the existing literature. Concerning the associations among the NIAS subscales [4,8,11,12,21] (see Appendix A), we expected the strongest correlations between the NIAS subscales Lack of Appetite and Fear of Negative Consequences of Eating, followed by the associations between Picky Eating and Lack of Appetite, and finally between Picky Eating and Fear of Negative Consequences of Eating.

Second, we examined the reliability of the NIAS subscales and total scale. Based on prior studies [4,8,11,12,21] (see Appendix A), we expected to find acceptable (0.80 > α/ω ≥ 0.70) to good (0.90 > α/ω ≥ 0.80) Alpha coefficients and McDonald’s omega coefficients for the NIAS Picky Eating and Lack of Appetite Food subscales, and good to excellent (α/ω ≥ 0.90) Alpha coefficients and McDonald’s omega coefficients for the NIAS Fear of Negative Consequences of Eating subscale and total scale.

Third, we examined the measurement invariance of NIAS across gender (male/female) and age [young adults (<40 years)/adults (≥40 years)] and BMI groups (Underweight/Healthy Weight/Overweight). Based on the existing literature, we expected to find scalar measurement invariance across gender [4,11] and weight groups [11]. We did not expect significant gender differences on the NIAS subscales [8,11]; whereas we expected that underweight individuals would score higher on the NIAS Lack of Appetite Scale compared to overweight individuals [4,8,11,12]. As no prior research has investigated measurement invariance across age groups in adult populations [young adults (<40 years)/adults (≥40 years)], no a priori hypotheses were formulated.

Finally, we examined the relations between the NIAS subscales and BMI, eating disorder symptomatology (i.e., Drive for Thinness, Bulimia, and Body Dissatisfaction), emotional disturbances (anxiety, depression), physical symptoms, identity dimensions (i.e., diffusion, synthesis) and self-esteem. Concerning BMI, we expected a significant negative association between the NIAS Lack of Appetite subscale and BMI [4,8,11,12]. The relationship between eating disorder symptomatology and the NIAS subscales was often non-significant, except in the study of Zickgraf and Ellis [8], who reported a positive association between the EAT-26 [22] and all NIAS scales, possibly due to the presence of individuals with ED in their sample. Similarly, Presseller et al. [23] found small to moderate associations between the EDE-Q subscales [10] and the NIAS scales in a sample of participants with an eating disorder. Additionally, Zickgraf & Ellis [8] also found positive associations between emotional undereating (Drive for Thinness) and all NIAS scales; and a negative association between emotional overeating (Bulimia) and Lack of Appetite, and a positive association with Fear of the Consequence of Eating. Based on DSM-5 ARFID criteria [2], we did not expect significant associations between Body Dissatisfaction and NIAS scales. With respect to emotional disturbances, we expected to find significant positive associations between anxiety and depression and all NIAS scales [4,8,11,12]. Additionally, based on previous research [7,8], we expected positive associations between physical complaints (and more specific gastrointestinal complaints) and all NIAS scales. Finally, based on established relations between identity and other disordered eating behaviors [24], we expected that NIAS scales would be positively associated with identity diffusion, and negatively associated with identity synthesis and self-esteem [24].

## 2. Materials and Methods

### 2.1. Participants & Procedure

The sample consisted of 425 adult participants from the Dutch-speaking community in Flanders (Belgium), of whom 424 completed the NIAS and were included in the study. Of these 424 participants, 74.8% (*n* = 317) identified as female, 24.8% (*n* = 105) as male, and 0.5% (*n* = 2) as “other.” The mean age of the sample was 36.76 years (SDage = 14.84 years; range = 18–65 years; 56.8% <40 years; 43.2% ≥40 years), and the mean BMI was 25.15 kg/m^2^ (SD_BMI_ = 5.11 kg/m^2^; range = 15.57–46.65 kg/m^2^; 4.2% underweight; 50.7% normal weight; 29% overweight; 15.6% obese; 0.5% missing). Participants were recruited via various online platforms by master’s thesis students and were invited to participate in a study on the associations between identity and psychological and somatic symptoms. Individuals who were invited and willing to participate could access the questionnaire through a link directing them to Pavlovia, which is the survey platform used in the present study. Before starting the questionnaire, participants were required to read and sign an informed consent form. After completing the questionnaires, participants were thanked for their participation and provided with information about the principal researchers and relevant helplines they can contact in case of emotional distress. The study was approved by the Social and Societal Ethics Committee of KU Leuven (Belgium) (protocol code G-2024-8530-R2(MIN) and date of approval 23 October 2024). The study was preregistered on the Open Science Framework (https://osf.io/fbc8s, accessed on 22 September 2026).

### 2.2. Instruments

#### 2.2.1. Avoidant/Restrictive Food Intake Disorder

ARFID symptoms were assessed using the Nine Item ARFID Screen (NIAS) [8], a 9-item self-report measure. Participants rated each item on a 6-point Likert scale ranging from 0 (strongly disagree) to 5 (strongly agree), with higher scores indicating more severe symptom severity. Example items are as follows: Picky Eating (e.g., ‘I’m a picky eater’), Fear (e.g., ‘I restrict myself to certain foods because I am afraid that other foods will case gastrointestinal discomfort, choking or vomiting’), and (Lack of) Appetite (e.g., ‘I am not very interested in eating, I seem to have a smaller appetite than other people’).

#### 2.2.2. Body Mass Index (BMI)

To assess participants’ BMI, participants reported their height in centimeters and weight in kilograms. We calculated BMI by dividing weight in kilograms by their squared length in meters [weight in kilograms/(length in meters)^2^].

#### 2.2.3. Eating Disorder Symptoms

To measure eating disorder symptoms, we administered the three subscales of the Eating Disorder Inventory-3 (EDI-3) [25,26], namely Drive for Thinness, Bulimia and Body Dissatisfaction. Each of the 25 items of the EDI-3 was rated on a six-point Likert scale ranging from 1 (never) to 6 (always). The Drive for Thinness subscale (n_items_ = 7) measures a desire to be thinner (e.g., ‘I’m thinking about going on a diet’; α = 0.91 in the present study). The Bulimia subscale (n_items_ = 8) assesses binge eating and purging (e.g., ‘I eat when I’m upset’; α = 0.86 in the present study). The Body Dissatisfaction subscale (n_items_ = 10) measures dissatisfaction with the shape and size of specific body parts (e.g., ‘I think my stomach is too fat’; α = 0.91 in the present study). The alpha coefficient of the EDI-3 eating disorder composite score (sum of DT, B and BD) was 0.94.

#### 2.2.4. Anxiety & Depressive Symptoms

The Symptom Checklist-90-Revised (SCL-90-R) [27] was applied to assess anxiety (16 items; ‘Feeling scared’; α = 0.92 in the present study) and depressive symptoms (10 items; ‘Worrying too much’; α = 0.95 in the present study). Participants rated the frequency of specific symptoms experienced during the past week on a 5-point Likert scale ranging from 1 (totally not) to 5 (very severe). Higher scores indicate higher levels of anxiety and depressive feelings.

#### 2.2.5. Somatic Symptoms

Somatic symptoms burden was assessed using the Patient Health Questionnaire-15 (PHQ-15) [28,29], a 15-item questionnaire evaluating the extent to which participants were bothered by a range of somatic symptoms (e.g., headache, shortness of breath, constipation, loose bowels, or diarrhea, nausea, gas or indigestion; α = 0.81 in the present study) during the past four weeks. Items were rated on a 3-point scale ranging from 0 (not bothered at all) to 2 (bothered a lot).

#### 2.2.6. Identity Functioning

Identity functioning was measured with the Erikson Psychosocial Stage Inventory (EPSI) [30,31], a 12 item self-report instrument. Participants responded to each item on a 5-point Likert scale ranging from 1 (strongly disagree) to 5 (strongly agree). Six items measure identity confusion (e.g., ‘I can’t decide what I want to do with my life’; α = 0.80 in the present study), and six items measure identity synthesis (e.g., ‘I’ve got a clear idea of what I want to be’; α = 0.74 in the present study).

#### 2.2.7. Self-Esteem

Finally, self-esteem was assessed with the Rosenberg Self-Esteem Scale [32], a 10 item self-report questionnaire. Participants rated each item (e.g., ‘I feel that I’m a person of worth’; α = 0.93 in the present study) on a 4-point Likert scale ranging from 1 (strongly disagree) to 4 (strongly agree). Item scores were averaged to obtain a mean score ranging from 1 to 4, with higher scores indicating higher levels of global self-esteem [32,33].

### 2.3. Analyses

Of the 425 participants, 424 were included in the analyses because one participant had missing data on the NIAS. Missing demographic data (BMI: *n* = 2; gender: *n* = 2) were not imputed and were handled using available-case analyses. For questionnaire measures, scale scores were calculated based on the available item responses, provided that at least 80% of the items were completed; no missing data imputation was performed.

First, we performed confirmatory factor analyses (CFAs) using Mplus version 8.8 [34] to test the three-factor structure of the Dutch version of the NIAS and a second-order model in which each of the subscale’s load on one higher-order latent factor (i.e., NIAS-total). Model parameters were estimated with Maximum likelihood estimator with Huber-White standard errors and scaled chi-square, given that sample size is reasonable and the number of response categories is 6 [35]. To investigate the robustness of the estimation algorithm, the one-factor and three-factor models (in the total sample) were re-estimated using the weighted least squares mean and variance adjusted (WLSMV) estimator. Three indices were used to evaluate model fit: (1) the Comparative Fit Index (CFI), with values between 0.90 and 0.95 indicating acceptable fit and values greater than 0.95 indicating good fit; (2) the Standardized Root Mean Square Residual (SRMR), with values below 0.08 indicating acceptable fit and values below 0.06 indicating good fit; and (3) the Root Mean Square Error of Approximation (RMSEA) with values below 0.08 indicating acceptable fit and values below 0.06 indicating good fit [36].

Second, we examined the reliability of the NIAS subscales using McDonald’s Omega and Cronbach’s alpha coefficients with JASP 0.96. Alpha and Omega coefficients exceeding 0.70, 0.80, and 0.90 were interpreted as indicative of acceptable, good, and excellent, respectively [37].

Third, we examined measurement invariance across gender, age (young adults aged 18–39 and adults aged 40–65 years) and BMI groups. Measurement invariance refers to the extent to which a questionnaire measures the same construct across different groups [38]. Configural invariance indicates that the same factor structure applies across groups (gender/age/BMI groups). Metric invariance demonstrates that the factor loadings are equivalent, allowing associations between constructs to be compared across groups. Scalar invariance additionally requires equivalent item intercepts, permitting meaningful comparisons of latent factor means [38]. Following Chen [38], measurement invariance was considered supported when changes in model fit remained within the recommended thresholds: ΔCFI ≤ 0.010, ΔRMSEA ≤ 0.015, and ΔSRMR ≤ 0.030 for metric invariance or ≤0.010 for scalar invariance. When scalar invariance across gender/age/BMI is obtained, mean differences between these groups can be interpreted meaningfully.

Finally, Pearson correlation coefficients were calculated to examine the associations between the NIAS subscales with BMI, eating disorder symptoms (i.e., Drive for Thinness, Bulimia, Body Dissatisfaction), emotional disturbances (anxiety and depression), physical symptoms, identity (synthesis, diffusion), and self-esteem. Correlations will be interpreted according to Cohen’s [39] guidelines, with coefficients of 0.10, 0.30, and 0.50 representing small, medium, and large effect sizes, respectively. Like previous studies [8], the correlations between the NIAS scales and other study variables were also calculated while controlling for the EDI-3 eating disorder composite score (sum of DT, B and BD), to control for ED symptomatology.

## 3. Results

### 3.1. Factor Structure of the Dutch Version of the NIAS

Table 1 presents the fit indices for the confirmatory factor analyses (CFAs). The proposed three-factor structure of the NIAS showed adequate fit, whereas the more parsimonious one-factor model did not. A higher-order model in which the three first-order factors loaded onto a single factor yielded identical fit indices to the correlated three-factor model (see Table 1). Similar results were obtained when re-estimating those models with a WLSMV estimation algorithm. Thus, the correlations among the three NIAS subfactors can be represented by a higher-order factor. However, because the two models are statistically equivalent, the fit of the higher-order model provides no additional evidence that a NIAS total score measures a single general latent dimension. Given the superior conceptual and statistical support for the three-factor structure of the NIAS, we recommend that future research primarily use and interpret the three subscale scores. The NIAS total score was included in the present study to facilitate comparison with the existing literature, in which the overall score has frequently been used.

Table 2 presents the standardized factor loadings of the items on each of the three NIAS subscales. All items demonstrated substantial factor loadings exceeding 0.67.

Table 3 displays the means (standard deviations) of the mean item scores of the NIAS scales, the correlations among the scales/latent constructs, and McDonald’s Omega and Cronbach’s Alpha coefficients of the scales. The strongest association (*r* = 0.43, medium effect size) was found between the Lack of Appetite and Fear of Negative Consequences of Eating subscales, followed by the association between Picky Eating and Lack of Appetite (*r* = 0.34, medium effect size), and between Picky Eating and Fear of Negative Consequences of Eating (*r* = 0.28, small effect size). All associations between the NIAS subscales and total score were strong (all *r* > 0.70, large effect size).

### 3.2. Reliability of the Subscales and Total Scale of the Dutch Version of the NIAS

Table 3 displays the McDonald’s Omega and Cronbach’s alpha coefficients for all NIAS scales. The findings indicate that all NIAS scales demonstrate good internal consistency coefficients (all coefficients > 0.80).

### 3.3. Measurement Invariance of the NIAS Across Gender, Age, & Weight Category

Measurement invariance was investigated for three variables: gender, age, and BMI. When investigating measurement invariance across gender (Table 4), the rows Configural, Metric, and Scalar should be considered first. Measurement invariance testing across gender showed substantial changes in fit indices from the configural to the metric model (ΔCFI = −0.043, ΔRMSEA = 0.015, ΔSRMR = 0.102), indicating that metric invariance was not supported. Additional decreases in fit were observed from the metric to the scalar model (ΔCFI = −0.021, ΔRMSEA = 0.001, ΔSRMR = 0.017), suggesting that scalar invariance was also not achieved. Therefore, only configural invariance was established across gender (see Table 4). Thus, all items loaded on the same factors in both groups, but the factor loadings and intercepts differed.

In a second step, we relaxed the constraints of the metric model by allowing the factor loadings of Items 6 and 7 to differ across gender groups. This model was compared with the original configural model, resulting in partial metric invariance (ΔCFI = 0.003, ΔRMSEA = −0.007, ΔSRMR = 0.014) (see Table 4). In a subsequent step, we examined whether scalar invariance would hold for this final partial metric model. Specifically, a model with equal intercepts across gender groups, but different factor loadings for Items 6 and 7, was fitted. This model was compared with the corresponding partial metric model shown in Table 4 (ΔCFI = −0.026, ΔRMSEA = 0.008, ΔSRMR = 0.020). The findings provided evidence for partial metric invariance but not for partial scalar invariance. The scales therefore have a relatively similar meaning across gender groups, but baseline scores differ between the groups.

Table 5 presents the observed means and variances of the mean item scores of the three NIAS subscales for men and women. Because scalar invariance across gender was not established, these means were not statistically compared. The descriptive differences should therefore not be interpreted as evidence of gender differences in the underlying constructs.

Table 6 displays the goodness-of-fit statistics for measurement invariance testing across age groups (<40 years; ≥40 years). The age groups were defined as younger adults (<40 years) and middle-aged/older adults (≥40 years), consistent with distinctions commonly used in developmental psychology research and to ensure sufficiently large subgroup sizes for meaningful analyses.

When investigating measurement invariance across age (Table 6), the rows Configural, Metric, and Scalar should be considered first. Given that none of the models reached an acceptable fit (see Table 6, row 1, 2, and 3), indicating that comparisons between age groups should be made with caution, as the meaning and baseline scoring of the NIAS may differ between age groups. Notably, the Satorra-Bentler scaled χ^2^ value was higher for the configural model than for the metric model, which is an unusual finding. This pattern was attributable to a substantially lower scaling factor for the configural model (0.7515) than for the metric model (1.4007). When the Satorra-Bentler scaled χ^2^ values are converted to their unscaled counterparts, the expected increase in χ^2^ from the configural (χ^2^ = 194.24 = 258.469 × 0.7515) to the metric model (χ^2^ = 238.39 = 170.193 × 1.4007) is observed. One possible explanation for this discrepancy is skewness in the data.

To investigate the source of model misfit, the model was fitted separately in each subgroup, and the results were compared. For the <40 years age group, an acceptable fit was obtained (CFI = 0.950, SRMR = 0.063, RMSEA = 0.075), whereas for the ≥40 years age group, a cross-loading of Item 7 (0.396) on the Lack of Appetite factor was required to obtain an acceptable fit (CFI = 0.939, SRMR = 0.056, RMSEA = 0.076).

Additionally, we tested the configural, metric and scalar measurement models with the cross-loading of item 7 on the Lack of Appetite scale. However, even the models including this cross-loadings did not reach metric invariance (ΔCFI = −0.042, ΔRMSEA = 0.016, ΔSRMR = 0.071) nor scalar invariance (ΔCFI = −0.028, ΔRMSEA = 0.004, ΔSRMR = 0.003) (see Table 6, row 4, 5, and 6).

Table 7 presents the observed means and variances of the mean item scores of the three NIAS subscales for each age group. Because scalar invariance across age groups was not established, the group means were not statistically compared.

For body mass index (BMI), our sample was divided into 3 subgroups: low or normal BMI (BMI < 25), overweight (25 ≤ BMI < 30), and obese (BMI ≥ 30). BMI data were missing for two participants resulting in a sample of 422 subjects. Given the small size of the underweight group (*n* = 18), we deviated from our preregistration and combined the underweight and normal-weight groups for the BMI invariance analyses. As a result, the findings do not permit conclusions regarding differences between underweight individuals and any other BMI group, and should therefore be interpreted with appropriate caution. When investigating measurement invariance across BMI (Table 8), the rows Configural, Metric, and Scalar should be considered first. Here again, the Satorra-Bentler scaled χ^2^ value was also higher for the configural model than for the metric model, albeit to a lesser extent. Specifically, the scaled χ^2^ for the configural model was 163.516 with a scaling factor of 1.2559, resulting in an unscaled χ^2^ of 205.360, whereas the metric model yielded a scaled χ^2^ of 162.885 with a scaling factor of 1.4463, corresponding to an unscaled χ^2^ of 235.581. Here, metric invariance was established (metric vs. configural: ΔCFI = 0.017, ΔRMSEA = −0.019, ΔSRMR = 0.026), and scalar invariance (scalar vs. metric: ΔCFI = −0.011, ΔRMSEA = −0.001, ΔSRMR = 0.006) was nearly reached (see Table 8). Relaxing the assumption of equal factor loadings for item 7, only in the obesity group, resulted in partial scalar invariance (scalar vs. metric with factor loading of item 7 free in the obese group: ΔCFI = −0.010, ΔRMSEA = −0.002, ΔSRMR = 0.007). For research purposes, certainly item 7 deserves extra attention as it seems to behave differently across several subsamples.

Overall, we found significant differences across BMI categories for the three NIAS subscales [Wilks’ Lambda = 0.967, *F*(6, 834) = 2.378, *p* = 0.028, partial *η*^2^ = 0.017] (see Table 9). Participants with a low/normal BMI scored significantly higher on Lack of Appetite than participants with overweight, with a small effect size.

### 3.4. Correlations Between the NIAS Scales and Eating Disorder Symptomatology, BMI, Anxiety/Depression, Physical Symptoms, Identity, and Self-Esteem

Finally, we correlated the NIAS scales with eating disorder symptoms, BMI, anxiety and depression, physical symptoms, identity functioning, and self-esteem (see Table 10). The findings showed small correlations between all NIAS scales and eating disorder symptomatology (Drive for Thinness, Bulimia, and Body Dissatisfaction). The association between Bulimia and Lack of Appetite was not significant. Additionally, BMI was significantly negatively related with Lack of Appetite and Fear of Negative Consequences of Eating, but not with Picky Eating. All NIAS scales were moderately positively associated with Anxiety and Depression, with the strongest correlations observed for Lack of Appetite and Fear of Negative Consequences of Eating. Physical symptoms, including gastrointestinal symptoms, were positively associated with all NIAS scales. Finally, all NIAS scales were significantly positively associated with Identity Diffusion and negatively associated with Identity Synthesis and Self-Esteem. After controlling for eating disorder symptomatology (ED composite score), the correlation pattern remained very similar, although the correlations generally decreased slightly. However, the correlations between BMI and all NIAS scales became significantly negative and increased in magnitude.

## 4. Discussion

The present study evaluated the psychometric properties of the Dutch version of the Nine-Item ARFID Screen (NIAS) in a Flemish adult community sample. Overall, the findings provide support for the reliability and validity of the Dutch NIAS in a young adult Flemish sample (<40 years). The original three-factor structure, representing Picky Eating, Lack of Appetite, and Fear of Negative Consequences of Eating, was confirmed.

### 4.1. Factor Structure & Correlations

The three factor-structure is consistent with the original validation of the NIAS by Zickgraf and Ellis [8], who developed the NIAS to assess the three main ARFID presentations; and the same three-dimensional structure has subsequently been replicated in different languages and adult populations [4,11,12,21]. Although the higher-order model provided an equivalent fit to the correlated three-factor model, retaining the three-factor model appears theoretically preferable; given that ARFID presentations are conceptually distinguishable and can occur either separately or in combination, and research has demonstrated differential clinical and psychological correlations for the three dimensions [7,8,9]. The NIAS total score was included solely to enable comparison with previous studies, in which the overall score has been commonly reported. All nine items showed substantial standardized factor loadings (ranging from 0.67 to 0.88), suggesting that the items were strong indicators of their respective ARFID dimensions.

The correlations between the three NIAS subscales were small to moderate, ranging from *r* = 0.28 to 0.43. The strongest correlation emerged between Lack of Appetite and Fear of Negative Consequences of Eating (*r* = 0.43), confirming findings from other studies in adults [4,8,11,12]. One possible explanation is that Fear of Negative Consequence of Eating (e.g., gastrointestinal discomfort, choking, or vomiting) may reduce appetite over time. Conversely, Lack of Appetite may increase attention towards unpleasant sensations associated with eating. The strong correlations between the NIAS subscales and total score (*r*’s > 0.70) are expected given that the total score incorporates the three subscales.

### 4.2. Reliability

Internal consistency was good to excellent for all NIAS scales, with both Cronbach’s alpha and McDonald’s omega exceeding 0.80, like the findings of prior studies [4,8,11,12,21].

### 4.3. Measurement Invariance Across Gender, Age, and Body Mass Index (BMI)

The measurement invariance findings indicate that comparisons across demographic (gender, age) could not be made and anthropometric (BMI) variables should be made cautiously. Across gender, configural invariance was supported, but full metric invariance was not initially established. After freeing the parameters of Items 6 and 7, partial metric invariance was obtained. This suggests that the three ARFID dimensions have comparable meanings across gender, although the extent to which some items represent the underlying constructs differs between men and women. These findings differ from studies reporting stronger invariance across gender (e.g., Medina-Tepal et al. [17] in Mexican youth; Serier et al. [4] in a US veteran sample).

The findings regarding age invariance (<40 years vs. ≥40 years) deserve particular attention. None of the initial invariance models comparing younger and older adults showed satisfactory fit. When examined separately, the original three-factor model showed acceptable fit among younger adults, whereas the model for older adults required a cross-loading of Item 7 on the Lack of Appetite factor. Even after allowing this cross-loading, metric invariance was not achieved. Given the lack of configural invariance across age groups and the cross-loading observed for Item 7 among older participants, future research should examine the age-related functioning of NIAS items through differential item functioning (DIF) analyses to determine whether the construct is measured equivalently across adulthood.

Finally, the findings were more favorable for BMI. Metric invariance was supported across participants with low/normal weight, overweight, and obesity, indicating that NIAS items generally represented the underlying constructs similarly across weight categories. Scalar invariance was nearly achieved, and freeing parameters associated with Item 7 in the obesity group resulted in partial scalar invariance. However, the relatively small size of some BMI subgroups, especially the underweight group, limited the statistical robustness of the measurement invariance analyses and necessitated the combination of the underweight and normal-weight groups. As a result, no conclusions can be drawn regarding potential differences between underweight individuals and the other BMI groups, and the findings should therefore be interpreted with caution. Notwithstanding, participants with low/normal BMI reported significantly higher Lack of Appetite scores than those with overweight, although the effect size was small. Similar findings have been reported by Fekih-Romdhane et al. [11] and He et al. [12]. This association is plausible because lack of appetite or interest in eating can be related to reduced energy intake and consequently body weight [7]. The recurrent non-invariance of Item 7 may reflect its heterogeneous content, combining multiple feared consequences of eating (e.g., choking, vomiting, or gastrointestinal discomfort) with avoidance behavior. In community samples, avoidance due to gastrointestinal discomfort may also reflect complaints unrelated to fear-based ARFID, although this explanation requires further investigation.

### 4.4. Associations Between ARFID and Psychological Symptoms

#### 4.4.1. Associations with Eating Disorder Symptoms & BMI

We found small positive correlations between the NIAS scales and Drive for Thinness and Bulimia. Consistent with this overlap, Presseller et al. [23] found elevated NIAS scores across several eating disorder groups in a large Swedish adult sample, particularly among individuals with current anorexia nervosa. Similarly, Burton Murray et al. [9] showed that although the NIAS subscales captured ARFID presentations, the NIAS alone was less successful in differentiating ARFID from other eating disorders. Whether ARFID, which often has its onset in childhood, increases the risk of later developing shape/weight-related eating disorders remains unclear [3], highlighting the need for longitudinal research. Contrary to our hypothesis, all three NIAS subscales were positively associated with Body Dissatisfaction. Although the DSM-5 [2] distinguishes ARFID from other eating disorders by specifying that restriction is not driven by weight or shape concerns, emerging evidence indicates that ARFID symptoms and body image concerns are not necessarily mutual exclusive and may co-occur [9,23]. Thus, the distinction between ARFID-related eating difficulties and body image concerns may be less clear-cut than current diagnostic criteria suggest.

NIAS scores were also related with BMI. Lack of Appetite and Fear of Negative Consequences of Eating were negatively correlated with BMI, whereas Picky Eating was negatively associated with BMI only after controlling for eating disorder pathology. Negative associations between BMI and ARFID symptoms, particularly Lack of Appetite, have also been reported in previous NIAS validation studies [8,11,12]. These associations are unsurprising, as lack of interest in eating and fear-based food avoidance may be related to reduced food intake. In contrast, the weaker association between Picky Eating and BMI may reflect that sensory-based food avoidance primarily restricts dietary variety without necessarily reducing caloric intake. Indeed, previous research has found inconsistent or no association between picky eating and BMI, particularly in adults [8].

#### 4.4.2. Associations with Anxiety and Depression

Additionally, all three NIAS scales were moderately associated with anxiety and depression, with especially strong association for Lack of Appetite and Fear of Negative Consequences of Eating, in line with findings of other studies [4,8,11,12]. These results correspond closely with the broader ARFID literature in which anxiety disorders are one of the most common psychiatric comorbidities of ARFID [6]. Similarly, Brownlow et al. [7] recently found that adults screening positive for ARFID were more than twice as likely to report clinically relevant anxiety and depression after controlling for age and gender. Anxiety may be related to food avoidance, particularly when there is no appetite or when eating is associated with feared negative consequences, whereas depressive symptoms may develop partly as a consequence of restrictive eating and the resulting withdrawal from or difficulties in social (eating) situations or vice versa [7,40].

#### 4.4.3. Associations with Physical Symptoms

Physical symptoms, including gastrointestinal symptoms, were also positively associated with all NIAS subscales. Recent adult population-based data [7,15,41] indicate that ARFID-positive screens are more common among individuals with disorders of gut–brain interaction and that ARFID symptoms are associated with greater somatic symptom burden. The present findings support the clinical importance of assessing ARFID when patients report gastrointestinal or other physical complaints or vice versa.

#### 4.4.4. Associations with Identity Functioning & Self-Esteem

A novel contribution of the current study concerns the association between ARFID and identity functioning. Across all three NIAS scales, higher ARFID symptoms were associated with greater Identity Diffusion and lower Identity Synthesis. As far as we know, the association between identity functioning and ARFID has received little direct empirical investigation. There is, however, substantial evidence linking identity difficulties to eating symptomatology [42]. Specifically, identity diffusion may increase vulnerability to eating disorder symptoms; whereas identity synthesis may serve as a protective factor [42]. Our findings extend the literature by suggesting that identity difficulties may also be related to ARFID. However, given that our data are cross-sectional in nature, longitudinal research is urgently needed to determine whether identity diffusion precedes the development of ARFID symptoms; develops as a consequence of persistent restrictive or picky eating, or whether reciprocal processes occur (as in anorexia and bulimia nervosa) [42]. The negative association between NIAS scores and self-esteem provided additional evidence that ARFID-symptoms are associated with broader self-functioning. Although research examining self-esteem in ARFID is also scarce, a study in Lebanese adults [43] identified self-esteem as a mediator in the association between perfectionism and ARFID symptomatology. These findings suggest that ARFID symptoms may be related to the individuals’ identity functioning and deserve more systematic investigation.

### 4.5. Strengths & Limitations

In sum, the present study evaluated multiple aspects of the psychometric functioning of the NIAS in a sample of Flemish adults, including factorial validity, internal consistency, measurement invariance across demographic/anthropometric characteristics and associations with external constructs. The inclusion of identity functioning and self-esteem extends previous research, which focused primarily on eating behavior, psychopathology and physical health. Several limitations should also be considered. First, the use of a convenience sample recruited through master’s thesis students via online platforms may have introduced selection bias, as the sample was heavily skewed toward women (74.8%) and younger adults, thereby limiting the representativeness of the study sample. Additionally, the subgroup sample sizes particularly within age and BMI categories, were smaller than the total sample and may have reduced the stability of measurement invariance analyses. This issue is particularly relevant given the differential item functioning of item 7. Second, the study relied exclusively on self-report measures, which can inflate the relations between the variables due to shared method variance. Therefore, future studies should apply a multi-method approach by objectively measuring anthropomorphic variables, observing eating behavior, or using other informants. Third, the cross-sectional nature of the study does not permit inferences regarding the directionality or causality of the associations between ARFID symptoms and psychological and somatic variables, such as anxiety, depression, identity functioning, self-esteem, and somatic symptoms (including gastrointestinal symptoms). Therefore, longitudinal studies are needed to clarify the directionality of the associations between these variables and to examine the co-development of these variables over time.

## 5. Conclusions

Overall, the Dutch version of the NIAS demonstrated a clear three-factor structure and good internal consistency and was associated with ED symptoms, BMI, psychological/physical distress and identity functioning in a Flemish sample. At the same time, the measurement invariance findings indicate that comparisons across demographic groups (gender, age) should not be made and comparisons across anthropomorphic (BMI) groups should be made cautiously. In particular, the recurrent differential functioning of Item 7 warrants further investigation in larger and more diverse samples.

## Figures and Tables

**Table 1:** Results of the confirmatory factor analyses on the Dutch version of the NIAS items.

Model	CFI	SRMR	Satorra-Bentler Scaled Χ^2^	Df	RMSEA	90% CI RMSEA
First-order one-factor model	0.437	0.147	596.068	27	0.223	0.208–0.239
First-order three-factor model	0.946	0.055	78.208	24	0.073	0.055–0.091
Second-order model with three NIAS subscales loading on one higher-order factor	0.946	0.055	78.208	24	0.073	0.055–0.091

NIAS = Nine Item ARFID Screen; CFI = Comparative Fit Index; SRMR = Standard Root Mean Square Residual; RMSEA = Root Mean Square Error of Approximation; 90% CI = 90% confidence interval.

**Table 2:** Nine Item ARFID Screen (NIAS) items [8] and factor loadings.

Items	Factor Loadings
Picky Eating	
1. I am a picky eater.	0.82
2. I dislike most of the foods that other people eat.	0.85
3. The list of foods that I like and will eat is shorter than the list of foods I won’t eat.	0.67
(Lack of) Appetite	
4. I am not very interested in eating; I seem to have a smaller appetite than other people.	0.81
5. I have to push myself to eat regular meals throughout the day, or to eat a large enough amount of food at meals.	0.84
6. Even when I am eating a food I really like, it is hard for me to eat a large enough volume at meals.	0.84
Fear of Negative Consequences of Eating	
7. I avoid or put off eating because I am afraid of gastrointestinal discomfort, choking, or vomiting.	0.88
8. I restrict myself to certain foods because I am afraid that other foods will cause gastrointestinal discomfort, choking, or vomiting.	0.77
9. I eat small portions because I am afraid of gastrointestinal discomfort, choking, or vomiting.	0.83

**Table 3:** Means (standard deviations) of the mean item scores of the NIAS scales, the correlations between the scales/latent constructs (second row), and McDonald’s Omega and Cronbach’s Alpha coefficients of the scales.

	M	(SD)	Correlations			McDonald’s ω	Cronbach’s α
			Picky Eating	Lack of Appetite	Fear		
Picky Eating	1.22	(1.21)	-	0.34 *** 0.37 ***	0.28 *** 0.28 ***	0.83	0.82
Lack of Appetite	1.00	(1.21)		-	0.43 *** 0.54 ***	0.87	0.86
Fear	0.64	(1.04)			-	0.86	0.86
Total	0.95	(0.87)	0.73 ***	0.80 ***	0.73 ***	0.90	0.83

*** *p* < 0.001.

**Table 4:** Goodness-of-fit indices for testing measurement invariance across gender.

	Satorra-Bentler Scaled Χ^2^	df	CFI	ΛCFI	RMSEA	Δ RMSEA	SRMR	ΔSRMR
Configural	110.541	48	0.937	-	0.079	-	0.064	-
Metric	162.860	57	0.894	−0.043	0.094	0.015	0.166	0.102
Scalar	192.679	66	0.873	−0.021	0.095	0.001	0.183	0.017
Metric, loadings on items 6 & 7 different across gender ^a^	115.048	55	0.940	0.003	0.072	−0.007	0.078	0.014
Scalar, loadings on items 6 & 7 different across gender ^b^	150.292	64	0.914	−0.026	0.080	0.008	0.098	0.02

^a^ The configural model serves as baseline for the metric model with loadings of items 6 and 7 different across gender; ^b^ the metric model with loadings on items 6 and 7 different across gender serves as baseline for this scalar model; *df* = degrees of freedom; CFI = Comparative Fit Index; RMSEA = Root Mean Square Standard Error of Approximation; SRMR = Standardized Root Mean Square Residual.

**Table 5:** Means (standard deviations) of the mean item scores of the NIAS subscales for females and males.

	Females (*n* = 317)		Males (*n* = 105)	
	M	(SD)	M	(SD)
Picky Eating	1.29	(1.23)	1.02	(1.14)
Lack of Appetite	1.11	(1.28)	0.66	(0.90)
Fear	0.72	(1.09)	0.39	(0.84)

**Table 6:** Goodness-of-fit indices for testing measurement invariance across age groups (<40 years; ≥40 years).

	Satorra-Bentler Scaled Χ^2^	df	CFI	ΛCFI	RMSEA	Δ RMSEA	SRMR	ΛSRMR
Configural	258.469	48	0.793	-	0.144	-	0.066	-
Metric	170.193	57	0.888	0.095	0.097	−0.047	0.137	0.071
Scalar	209.009	66	0.859	−0.029	0.101	0.004	0.143	0.006
Configural with cross-loading of item 7 on Lack of Appetite	104.528	46	0.942	-	0.077	-	0.060	-
Metric with cross-loading of item 7 ^a^	156.028	55	0.900	−0.042	0.093	0.016	0.131	0.071
Scalar with cross-loading of item 7 ^b^	194.835	65	0.872	−0.028	0.097	0.004	0.134	0.003

^a^ The configural model with cross-loading of item 7 on Lack of Appetite serves as baseline for the metric model with cross-loading of item 7 on Lack of Appetite; ^b^ the metric model with cross-loading of item 7 on Lack of Appetite serves as baseline for the scalar model with cross-loading of item 7 on Lack of Appetite; *df* = degrees of freedom; CFI = Comparative Fit Index; RMSEA = Root Mean Square Standard Error of Approximation; SRMR = Standardized Root Mean Square Residual.

**Table 7:** Means (standard deviations) of the mean item scores of the NIAS subscales for young adults (<40 years) and adults (≥40 years).

	Young Adults (<40 Years) (*n* = 241)		Adults (≥40 Years) (*n* = 183)	
	M	(SD)	M	(SD)
Picky Eating	1.32	1.24	1.09	1.17
Lack of Appetite	1.17	1.32	0.78	1.03
Fear	0.77	1.18	0.46	0.78

**Table 8:** Goodness-of-fit indices for testing measurement invariance across BMI categories (low/normal; overweight; obese).

	Satorra-Bentler Scaled Χ^2^	df	CFI	ΛCFI	RMSEA	Δ RMSEA	SRMR	ΛSRMR
Configural	163.516	72	0.914	-	0.095	-	0.067	-
Metric	162.885	90	0.931	0.017	0.076	−0.019	0.093	0.026
Scalar	192.534	108	0.920	−0.011	0.075	−0.001	0.099	0.006
Metric, factor loading of item 7 free in obese group ^a^	158.744	89	0.934	0.020	0.075	−0.020	0.090	0.023
Scalar, Factor loading of item 7 free in obese group ^b^	187.789	107	0.924	−0.010	0.073	−0.002	0.097	0.007

^a^ The configural model serves as baseline for the metric model with the factor loading of item 7 free in the obese group; ^b^ the metric model with the factor loading of item 7 free in the obese group serves as baseline for the scalar model with the factor loading of item 7 free in the obese group; *df* = degrees of freedom; CFI = Comparative Fit Index; RMSEA = Root Mean Square Standard Error of Approximation; SRMR = standardized Root Mean Square Residual.

**Table 9:** Means (standard deviations) of the mean item scores of the NIAS subscales for the three BMI categories.

	Group 1 Low (*n* = 18)/Normal Weight (*n* = 215) (*n* = 233)		Group 2 Overweight (*n* = 123)		Group 3 Obesity (*n* = 66)				
	M	(SD)	M	(SD)	M	(SD)	F(2, 419)	Partial ή^2^	Post-Hoc Tests
Picky Eating	1.29	(1.25)	1.09	(1.11)	1.18	(1.23)	1.146	0.005	
Lack of Appetite	1.18	(1.28)	0.73	(1.01)	0.88	(1.25)	6.183 **	0.029	2 < 1
Fear	0.72	(1.08)	0.57	(0.97)	0.45	(0.92)	2.021	0.010	

** *p* < 0.01. The reference values for effect sizes for Partial Eata Squared: small effect = 0.01; medium effect = 0.06; and large effect = 0.14.

**Table 10:** Correlations between the NIAS scales and eating disorder symptoms, BMI, anxiety/depression, physical symptoms, identity, and self-esteem (second row: correlations controlled for EDI-3 composite score).

	Picky Eating	Lack of Appetite	Fear	Total
EDI-3 ^a^				
Drive for Thinness	0.12 *	0.18 ***	0.18 ***	0.21 ***
Bulimia	0.11 *	−0.03	0.13 **	0.09
Body Dissatisfaction	0.17 ***	0.11 *	0.13 **	0.18 ***
BMI	−0.07	−0.19 ***	−0.12 *	−0.17 ***
	−0.15 **	−0.26 ***	−0.21 ***	−0.28 ***
SCL-90				
Anxiety	0.18 ***	0.46 ***	0.37 ***	0.45 ***
	0.14 **	0.45 ***	0.34 ***	0.41 ***
Depression	0.25 ***	0.50 ***	0.31 ***	0.47 ***
	0.20 ***	0.50 ***	0.26 ***	0.43 ***
PHQ-15				
Total	0.23 ***	0.42 ***	0.36 ***	0.44 ***
	0.18 ***	0.41 ***	0.32 ***	0.41 ***
Item 12. Constipation, loose bowels, or diarrhea.	0.13 **	0.12 *	0.27 ***	0.22 ***
	0.09	0.09	0.23 ***	0.18 ***
Item 13. Nausea, gas, or indigestion.	0.17 ***	0.29 ***	0.41 ***	0.37 ***
	0.12 *	0.26 ***	0.38 ***	0.33 ***
EPSI				
Identity Diffusion	0.27 ***	0.38 ***	0.26 ***	0.41 ***
	0.23 ***	0.37 ***	0.22 ***	0.37 ***
Identity Synthesis	−0.27 ***	−0.32 ***	−0.21 ***	−0.36 ***
	−0.23 ***	−0.30 ***	−0.16 ***	−0.32 ***
RSES	−0.26 ***	−0.39 ***	−0.26 ***	−0.40 ***
	−0.21 ***	−0.38 ***	−0.21 ***	−0.36 ***

^a^ Correlations between the EDI-3 scales and the NIAS scales were calculated as zero-order correlations; in contrast, correlations between the NIAS scales and the other measures were additionally examined while controlling for the EDI-3 Composite score, following previous research, with these partial correlations reported in the second row; EDI-3 = Eating Disorder Inventory-3 (*n* = 424), BMI = Body Mass Index (*n* = 422); SCL-90-R = Symptom Checklist-90-Revised (*n* = 424); PHQ-15 = Patient Health Questionnaire-15 (*n* = 424); EPSI = Erikson Psychosocial Stage Inventory (*n* = 421); RSES = Rosenberg Self-Esteem Scale (*n* = 423). * *p* < 0.05, ** *p* < 0.01, *** *p* < 0.001.

## Data Availability

Data are available upon request at laurence.claes@kuleuven.be.

## Appendix A

**Table A1:** Overview of Psychometric Characteristics of the NIAS in Adult Samples.

Authors Samples	Psychometric Features	Picky Eating	(Lack of) Appetite	Fear	Total Score
	**Internal Consistency**				
Zickgraf & Ellis (2018) [8]					
**S1**: 505 US adults/parents	α	0.87	0.87	0.93	0.90
69.5% females	ω	0.87	0.87	0.93	0.95
*M*_age_ = 36.6 (*SD* = 8.6)					
**S2**: 455 US clinical sample (ED)	α	0.86	0.87	0.91	0.88
48.6% females	ω	0.86	0.87	0.91	0.94
*M*_age_ = 33.6 (*SD* = 9.5)					
**S3**: 311 undergraduates	α	0.82	0.77	0.86	0.79
68.6% females	ω	0.83	0.78	0.86	0.89
*M*_age_ = 19.6 (*SD* = 2.5)					
He et al. (2021) [12]	α	0.74	0.73	0.86	0.86
1069 college students from China					
52.6% females					
*M*_age_ = 20.11 (*SD* = 1.01)					
Fekih-Romdhane et al. [11]	ω	0.75	0.80	0.90	0.88
515 Lebanese community adults					
69.9% females					
*M*_age_ = 27.55 (*SD* = 10.92)					
Serier et al. (2025) [4]					
1486 U.S. military veterans	α	0.87	0.89	0.90	0.86
55.2% females	ω	0.88	0.89	0.90	0.84
*M*_age_ = 29.3 (*SD* = 8.3)					
Breiner et al. (2025) [21]	α				
248 autistic adults (USA)	ASS	0.83–0.88	0.83–0.88	0.83–0.88	-
67.76% females					
*M*_age_ = 31.19 (*SD* = 5.60)					
389 general sample (USA)	HC	0.86–0.90	0.86–0.90	0.86–0.90	-
? females					
*M*_age_ = 37.42 (*SD* = 12.84)					
	**Test-Retest Reliability**				
Zickgraf et al. (2018) [8]	2 weeks				
Undergraduates	ICC	0.76	0.70	0.62	0.65
He et al. (2021) [12]	4 weeks				
	*r*	0.69	0.62	0.65	0.72
	**Correlations between scales**				
Zickgraf & Ellis (2018) [8]	Picky Eating	-			0.80
**S1** (adults)	(Lack of) Appetite	0.51	-		0.85
	Fear	0.42	0.58	-	0.85
Zickgraf & Ellis (2018) [8]	Picky Eating	-			
**S2** (clinical)	(Lack of) Appetite	0.55	-		
	Fear	0.30	0.47	-	
Zickgraf & Ellis (2018) [8]	Picky Eating	-			0.70
**S3** (undergraduates)	(Lack of) Appetite	0.43	-		0.77
	Fear	0.13	0.26	-	0.68
He et al. (2021) [12]	Picky Eating	-			-
	(Lack of) Appetite	0.76	-		-
	Fear	0.46	0.78	-	-
Fekih-Romdhane et al. (2023) [11]	Picky Eating	-			0.79
	(Lack of) Appetite	0.52	-		0.87
	Fear	0.49	0.66	-	0.86
Serier et al. (2025) [4]	Picky Eating	-			-
	(Lack of) Appetite	0.42	-		-
	Fear	0.31	0.56	-	-
	**Gender**				
Zickgraf & Ellis (2018) [8]	M/FM	ns	ns	ns	ns
He et al. (2021) [12]	M/FM	ns	M > FM	ns	ns
Fekih-Romdhane et al. (2023) [11]	M/FM	ns	ns	ns	ns
	**BMI**				
Zickgraf & Ellis (2018) [8]	*r*	ns	−0.15 *	ns	−0.10 *
Fekih-Romdhane et al. (2023) [11]	2 groups	ns	UW/HW > OW	ns	ns
He et al. (2021) [12]	3 groups	UW > HW > OW	UW > HW, OW	ns	UW > HW, OW
He et al. (2021) [12]	β	−0.09 *	−0.11 *	ns	−0.15 **
Serier et al. (2025) [4]	*r*	ns	−0.25 **	ns	-
	**Eating Disorders**				
Zickgraf & Ellis (2018) [8]	EAT-26	0.24 **	0.26 **	0.24 **	0.31 **
He et al. (2021) [12]	EAT-26	ns	ns	ns	-
Fekih-Romdhane et al. (2023) [11]	EAT-7	ns	ns	0.11 *	ns
Serier et al. (2025) [4]	EDE-Q Total	0.15 **	ns	0.17 **	-
	**Emotional Distress**				
Fekih-Romdhane et al. (2023) [11]	DASS-8	0.22 **	0.19 **	0.21 **	0.25 **
He et al. (2021) [12]	K10 (β)	ns	0.23 **	0.20 **	0.39 **
Serier et al. (2025) [4]	DASS21-depression	0.15 *	0.31 **	0.25 **	-
	DASS-21-anxiety	0.19 **	0.29 **	0.31 **	-
	DASS-21-stress	0.15 **	0.29 **	0.23 **	-
Zickgraf & Ellis (2018) [8]	DASS21-depression	0.13 **	0.26 **	0.23 **	0.27 **
	DASS-21-anxiety	0.16 **	0.30 **	0.34 **	0.34 **
	**GI symptoms**				
Zickgraf & Ellis (2018) [8]	VSI	0.32 **	0.42 **	0.69 **	0.61
	**Measurement Invariance**				
Zickgraf & Ellis (2018) [8]	Scalar measurement invariance between the **analogue clinical sample** and the **undergraduate sample** was supported.				
Fekih-Romdhane et al. (2023) [11]	Multi-group analyses demonstrated measurement invariance by **sex** (males vs. females) and **weight groups** (underweight/healthy weight vs. overweight/obese) at the configural, metric, and scalar level.				
He et al. (2021) [12]	Strong measurement invariance (configural, metric, and scalar) between college samples of the **United States** (*n* = 1533) and **China** (*n* = 1069) was supported.				
Serier et al. (2025) [4]	The NIAS was invariant at the configural, metric, and scalar levels for **self-identified gender**, **race and ethnicity**, and **sexual and gender minority identity**.				
Breiner et al. (2025) [21]	Strong measurement invariance (configural, metric, and scalar) on the NIAS between a sample of **autistic adults** and a comparison **general sample** was supported.				

EAT = Eating Attitude Test; DASS = Depression, Anxiety, Stress Scale; K10 = Kessler Psychological Distress Scale; VSI = Visceral Sensitivity Index (gastrointestinal (GI) symptom-specific anxiety). * *p* < 0.05, ** *p* < 0.01.
